# Supplementary material for: Simultaneous two-photon imaging of action potentials and subthreshold inputs in vivo
Source: Nat Commun. 2021 Dec 10;12:7229. doi: 10.1038/s41467-021-27444-9 (PMC8664861; doi:10.1038/s41467-021-27444-9)
Supplement: Supplementary file 1 — Supplementary Information [file 41467_2021_27444_MOESM1_ESM.pdf]

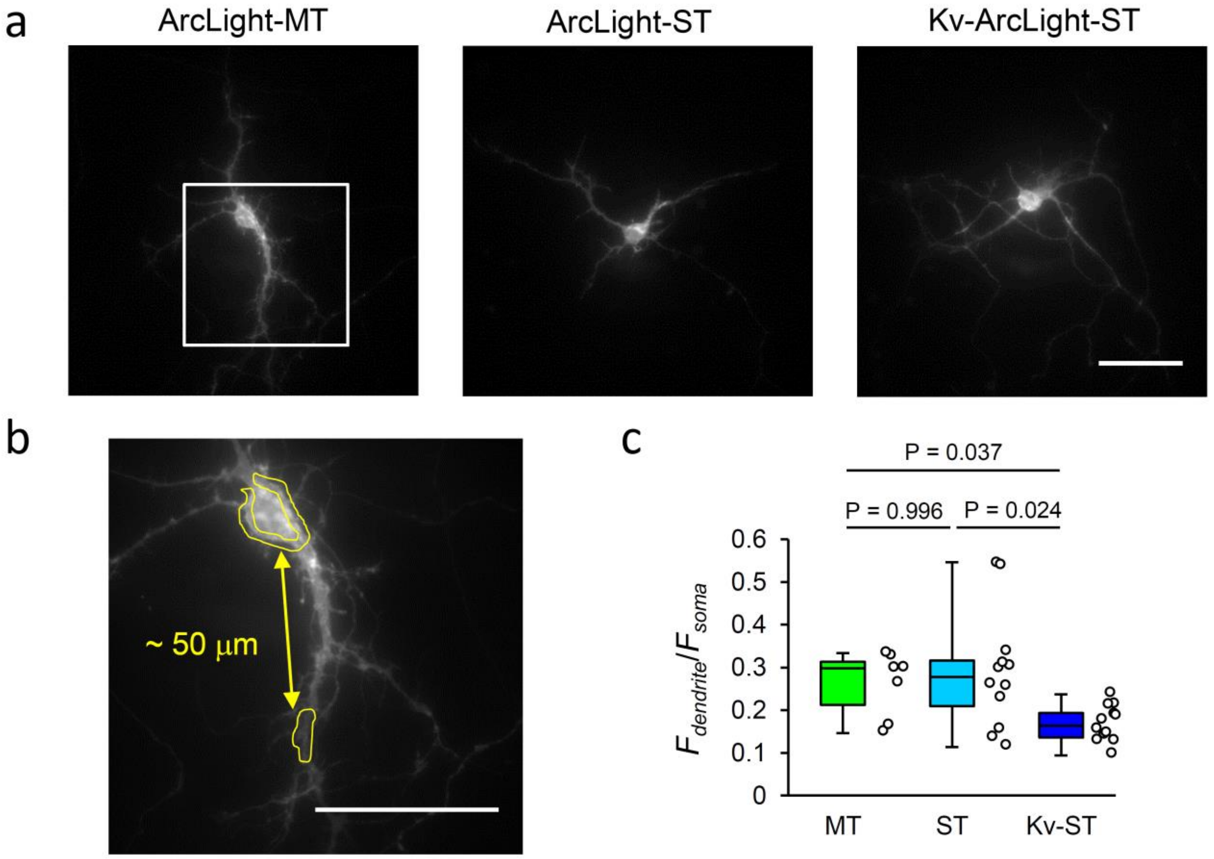

Supplementary Figure 1 | Expression patterns of ArcLight variants in cultured hippocampal neurons. (**a**) Representative images of neurons expressing ArcLight variants in vitro. Scale bar, 50 μm. (**b**) An example of analysis of somal localization of ArcLight variants. Average fluorescence in the soma and the dendrite were measured, and ratio between fluorescence in dendrite and soma was calculated. The membrane region of the soma and dendrites which is about 50 μm away from the soma was chosen for analysis. An image is the square region of the ArcLight-MT image in **a**. Scale bar, 50 μm. (**c**) Ratio between fluorescence in the dendrite and in soma. $F_{dendrite}/F_{soma}$ of ArcLight-ST was significantly smaller than other ArcLight variants. n = 7 cells (ArcLight-MT), 12 (ArcLight-ST and Kv-ArcLight-ST). Steel-Dwass test was performed for statistical analysis. On each box, the central line represents median, and the bottom and the top edges of the box represent 25th and 75th percentile, respectively. The lower and the upper whiskers extend to the minimum and the maximum data points, respectively.

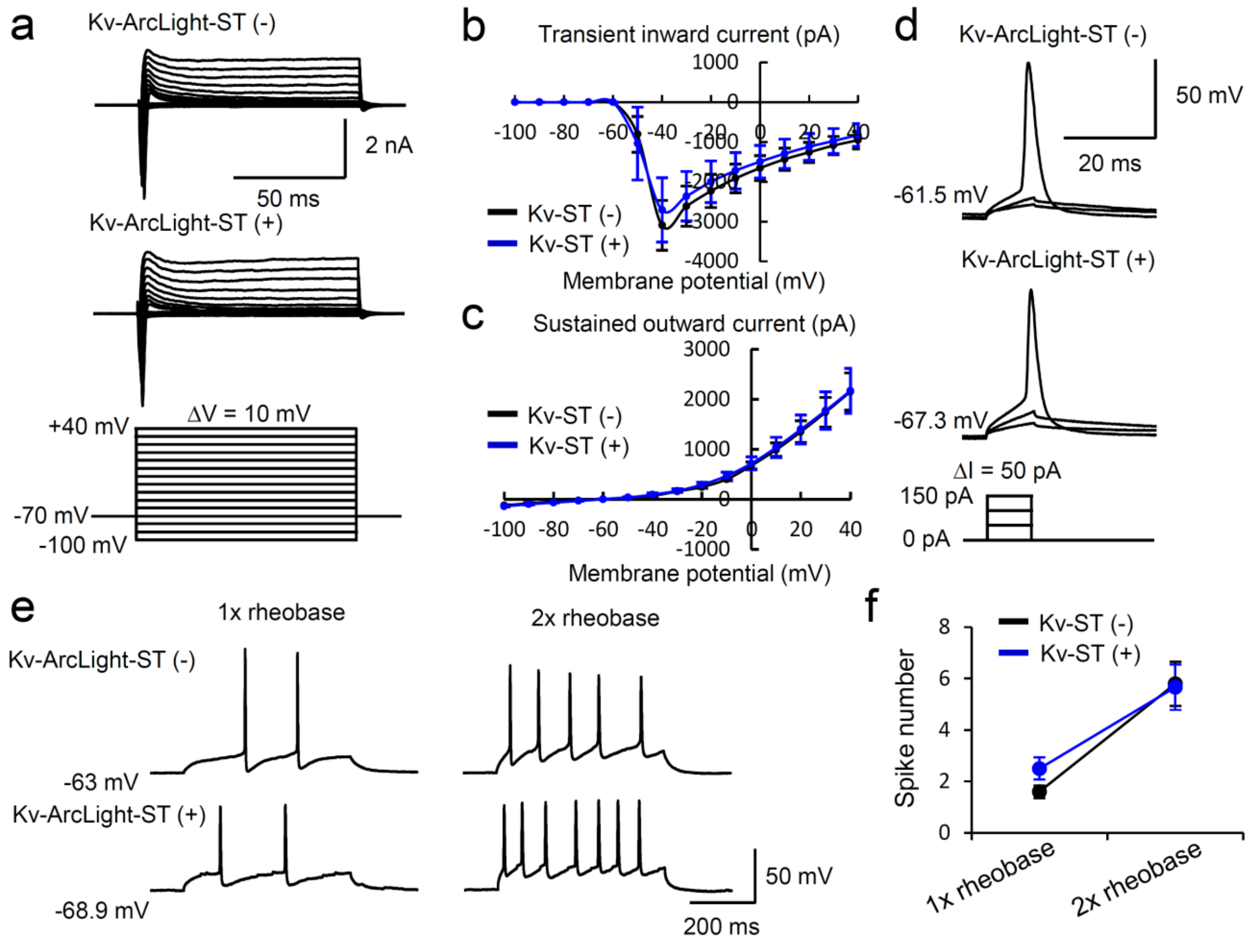

g

	Kv-ArcLight-ST (-)	Kv-ArcLight-ST (+)	P value
Capacitance (pF)	29.1 ± 5.3 (*n* = 10)	34.0 ± 6.2 (*n* = 9)	0.551
Input resistance (MΩ)	333.7 ± 22.0 (*n* = 10)	306.3 ± 27.1 (*n* = 9)	0.453
Peak Inward current (pA)	-3094.2 ± 622.8 (*n* = 10)	-2707.5 ± 807.8 (*n* = 9)	0.710
Outward current at +40 mV (pA)	2155.2 ± 371.6 (*n* = 10)	2173.0 ± 450.6 (*n* = 9)	0.976
Resting potential (mV)	-67.0 ± 2.2 (*n* = 5)	-64.4 ± 2.4 (*n* = 6)	0.445
Amplitude of action potentials (mV)	82.8 ± 7.0 (*n* = 5)	78.5 ± 4.2 (*n* = 6)	0.649
Half width of action potentials (ms)	2.44 ± 0.20 (*n* = 5)	2.85 ± 0.26 (*n* = 6)	0.250
Threshold (mV)	-41.2 ± 1.2 (*n* = 5)	-40.9 ± 4.0 (*n* = 6)	0.944
Rheobase for 500 ms-current injection (pA)	32.0 ± 3.7 (*n* = 5)	28.3 ± 1.7 (*n* = 6)	0.408
Spike number at 1x rheobase	1.60 ± 0.24 (*n* = 5)	2.50 ± 0.43 (*n* = 6)	0.107
Spike number at 2x rheobase	5.80 ± 0.86 (*n* = 5)	5.67 ± 0.88 (*n* = 6)	0.916

Supplementary Figure 2 | Electrical properties of neurons were not changed by expression of Kv-ArcLight-ST. (**a**) Representative current response to hyper and depolarizing voltage steps. Voltage-clamp was performed in the cultured hippocampal neurons. Holding potential was -70 mV. (**b**) Voltage-dependent transient inward current. (**c**) Voltage-dependent sustained outward current. (**d**) Representative voltage response to short depolarizing current steps of 10 ms recorded with current-clamp mode. (**e**) Representative voltage response to long current steps of 500 ms. (**f**) Firing property of Kv-ArcLight-ST-positive and negative neurons. (**g**) Passive and active electrical properties of Kv-

ArcLight-ST-positive and negative neurons. Mean ± SEM is presented. Mann-Whitney test was used for statistical analysis.

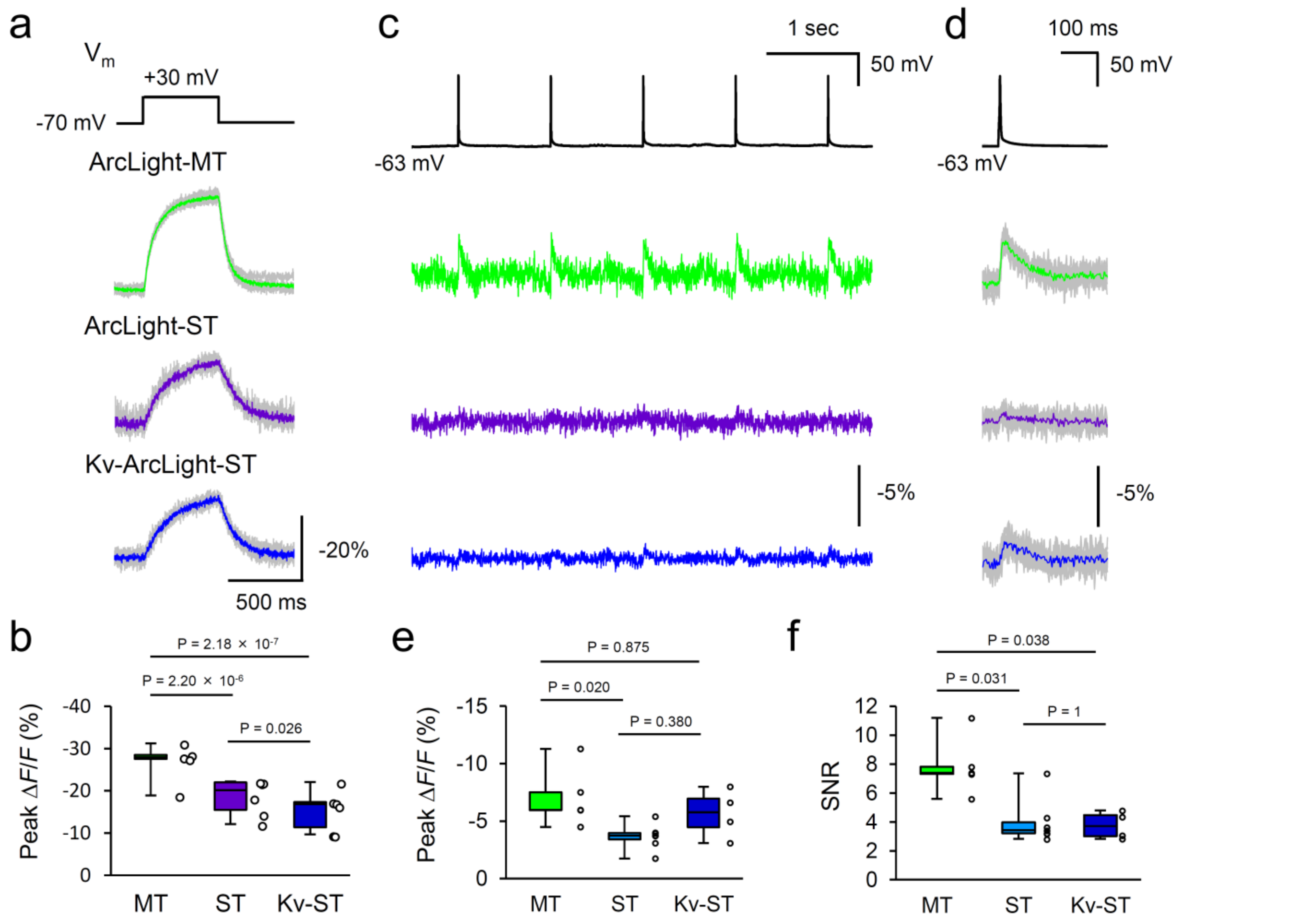

Supplementary Figure 3 | Detection of action potentials with ArcLight variants *in vitro*. (**a**) Response to large depolarizing voltage steps of 100 mV. Voltage steps were applied with voltage-clamp mode. (**b**) Peak fluorescence change in response to depolarizing steps of 100 mV. *n* = 5 cells (ArcLight-MT), 6 (ArcLight-ST and Kv-ArcLight-ST). (**c**) Representative dynamics of membrane potential and fluorescence of ArcLight variants. Action potentials were induced with current injection for 5 ms. Imaging was performed at 1 kHz, and low-pass-filtered at 200 Hz. (**d**) Averaged electrical (black) and optical (colored) signals over 10 action potentials. Individual trials are shown with gray. (**e, f**) Peak fluorescence change (**e**) and signal-to-noise ratio (**f**) in response to single action potential. *n* = 5 cells (ArcLight-MT), 8 (ArcLight-ST), 4 (Kv-ArcLight-ST). Data was collected from 2 ~ 3 batches of culture. Steel-Dwass test was used for statistical test. Excitation wavelength was 460 ~ 500 nm, excitation power was ~5.5 mW (~ 36 mW/mm^2) at the stage. On each box in (b), (e), and (f), the central line represents median, and the bottom and the top edges of the box represent 25th and 75th percentile, respectively. The lower and the upper whiskers extend to the minimum and the maximum data points, respectively.

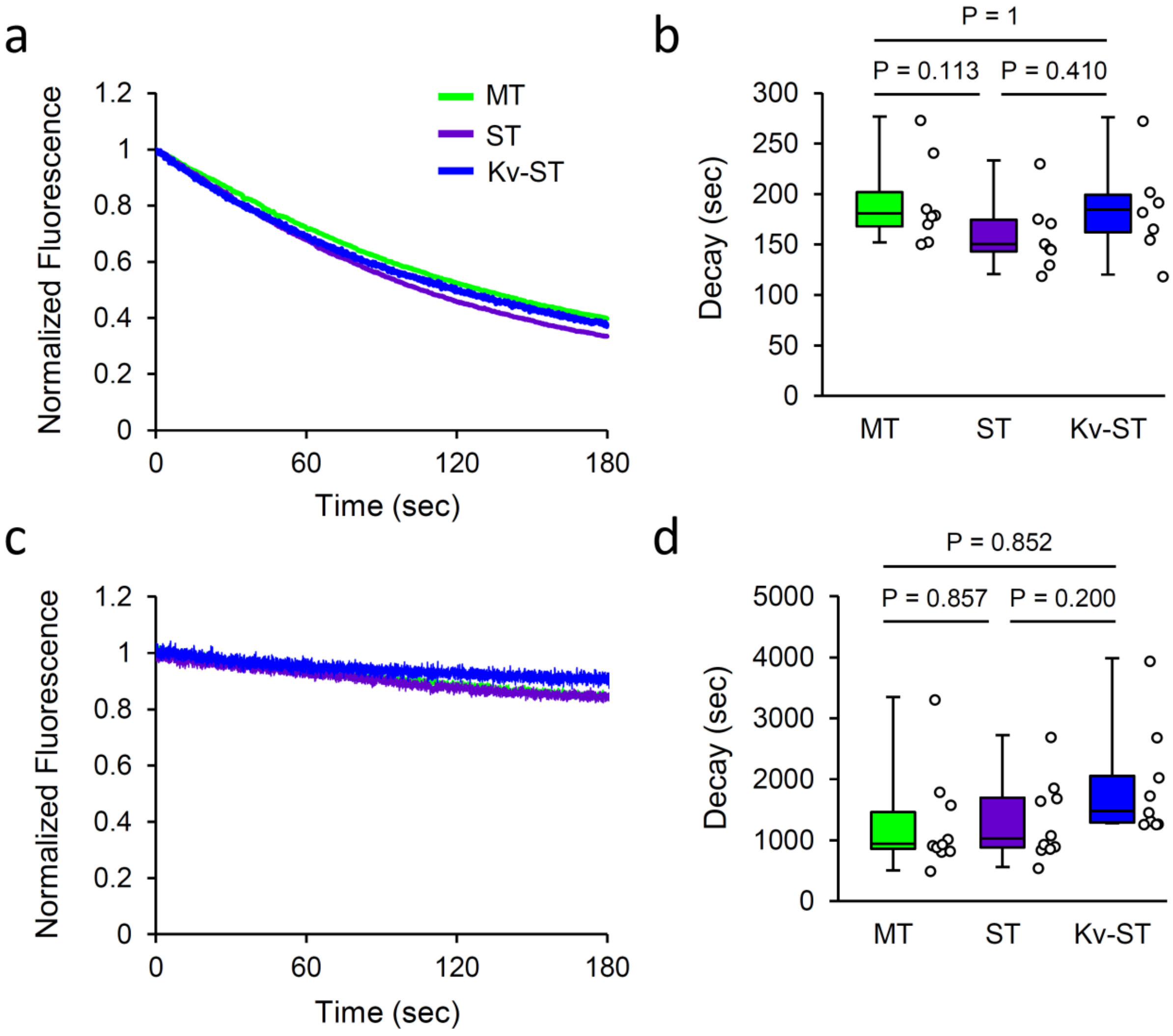

Supplementary Figure 4 | Photostability of ArcLight variants *in vitro*. (**a**) One-photon photobleaching kinetics of ArcLight variants. Cultured neurons expressing ArcLight-MT (*n* = 8 cells), ArcLight-ST (*n* = 8), Kv-ArcLight-ST (*n* = 7) were continuously illuminated with mercury arc lamp. Excitation wavelength was 460 ~ 500 nm, excitation power was ~5.5 mW (~ 36 mW/mm^2) at the stage. (**b**) Time constant of one-photon photobleaching. (**c**) Two-photon photobleaching kinetics of ArcLight variants. Cultured neurons expressing ArcLight-MT (*n* = 8 cells), ArcLight-ST (*n* = 8), Kv-ArcLight-ST (*n* = 7) were continuously scanned at 30 Hz with resonant scanning mode. Excitation power was 28 mW (~0.11 μW/pixel) at the stage. (**d**) Time constant of two-photon photobleaching. Steel-Dwass test was performed for statistical analysis. Data was collected from 3 different culture preparations in each experiment. On each box in (b) and (d), the central line represents median, and the bottom and the top edges of the box represent 25th and 75th percentile, respectively. The lower and the upper whiskers extend to the minimum and the maximum data points, respectively.

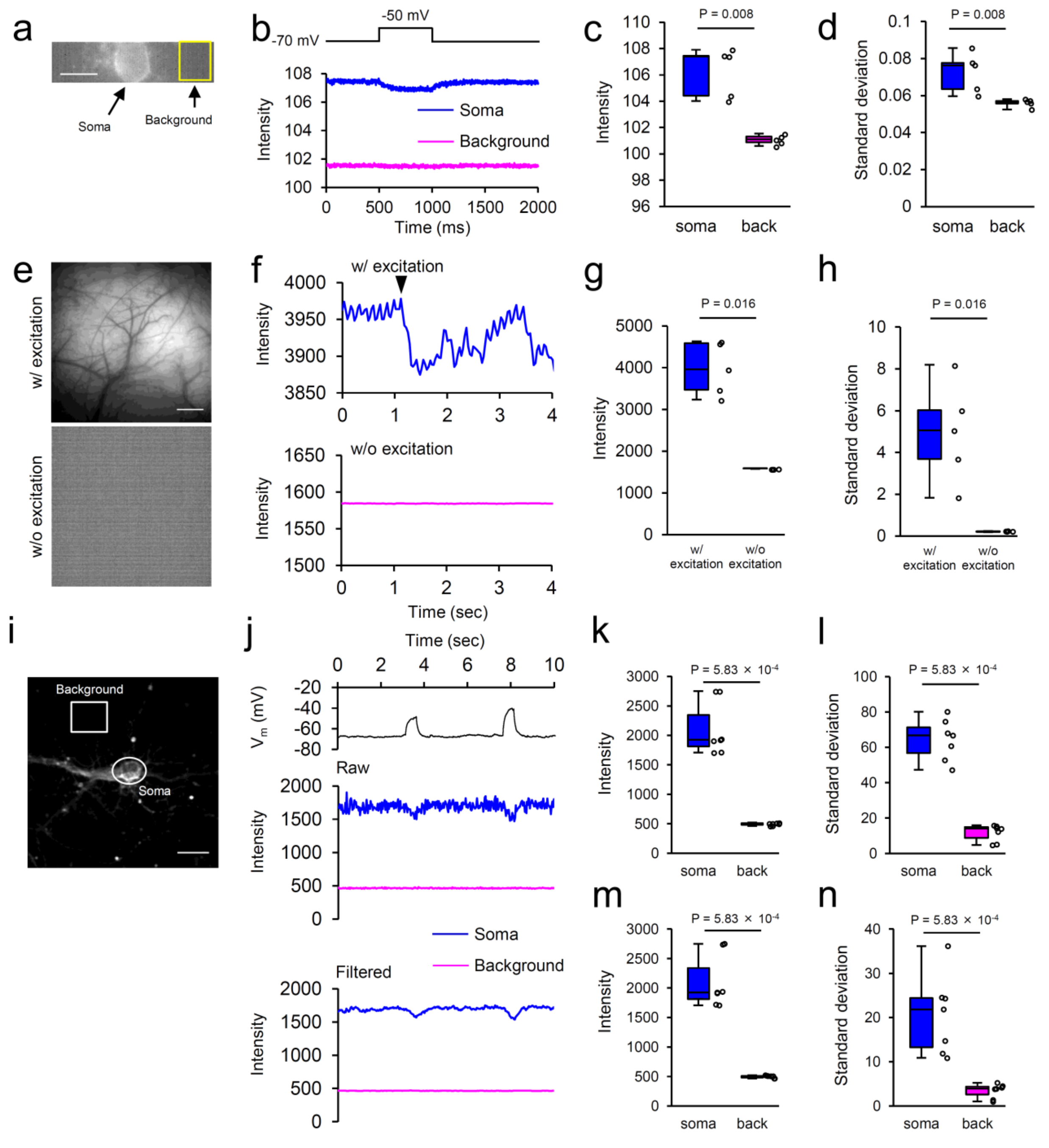

Supplementary Figure 5 | Effect of noise in our imaging systen. (**a-d**) One-photon imaging with Kv-ArcLight-ST at 1 kHz. (**a**) Cultured neurons expressing Kv-ArcLight-ST under the CMV promoter. Images were acquired with HC Image software, and were converted to 16-bit tiff file. A rectangle indicates the background of similar area of the soma. Scale bar, 10 μm. (**b**) Representative optical traces in the soma and in the background area. A depolarizing voltage step of 20 mV was applied. (**c, d**) Mean intensity (**c**) and standard deviation (**d**) of the baseline optical signals. Statistical analysis was performed using Mann-Whitney test. Data in Fig. 1 were analyzed. (**e-h**) One-photon wide-field imaging at 30 Hz *in vivo*. (**e**) Primary visual cortex expressing Kv-ArcLight-ST under the CAG promoter. Images were obtained with excitation light of 470 nm (~5.5 mW) (upper panel) or without excitation light (lower panel). Images were acquired with HC Image software, and were

converted to 16 bit tiff file. Scale bar, 500 μm. (**f**) Representative optical traces with or without excitation light. An arrowhead indicates the timing of visual stimulation. (**g, h**) Mean intensity (**c**) and standard deviation (**d**) of the baseline optical signals. Statistical analysis was performed using Mann-Whitney test. Data in Fig. 2 were analyzed. (**i-n**) Two-photon imaging with Kv-ArcLight-ST at 30 Hz *in vitro*. (**i**) Cultured neurons expressing Kv-ArcLight-ST under the CMV promoter. Images were acquired as 16-bit tiff file with Prairie system. A rectangle indicates the background of similar area of the soma. Scale bar, 20 μm. (**j**) Representative optical traces in the soma and in the background area. Depolarizing current steps were applied. (**k-n**) Mean intensity (**k,m**) and standard deviation (**l,n**) of the baseline optical signals. Statistical analysis was performed using Mann-Whitney test. Data in Fig. 5 were analyzed. On each box in (c), (d), (g), (h) and (k) ~ (n), the central line represents median, and the bottom and the top edges of the box represent 25th and 75th percentile, respectively. The lower and the upper whiskers extend to the minimum and the maximum data points, respectively.

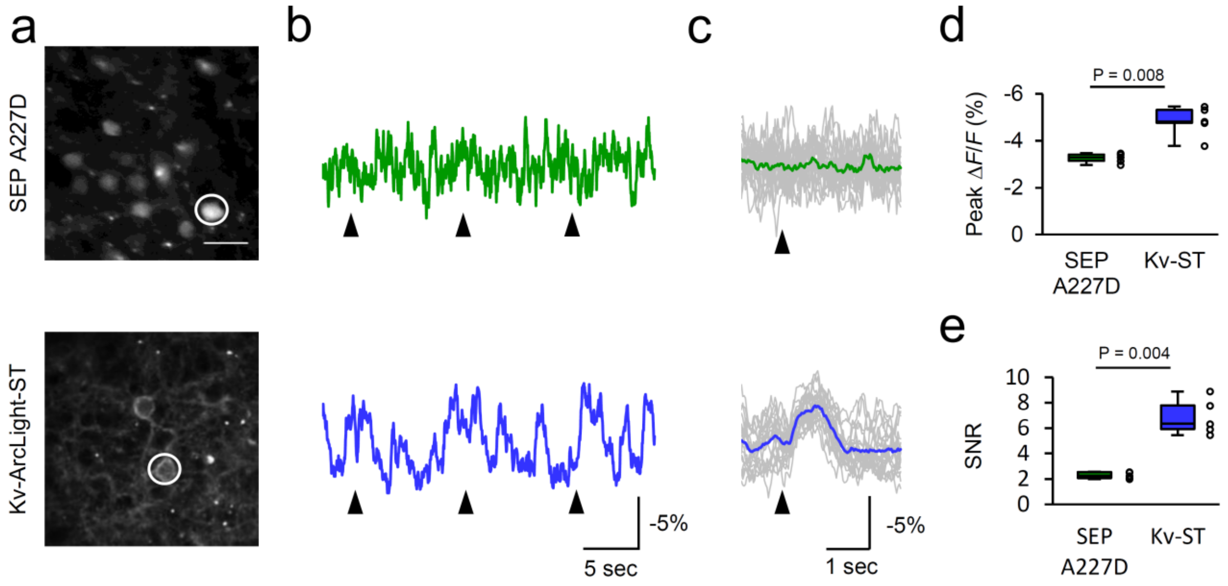

Supplementary Figure 6 | Change of cytosolic pH was not observed *in vivo*. (**a**) Two-photon image of layer 2/3 neurons expressing Super Ecliptic pHluorin (SEP) A227D without voltage-sensing domain (upper panel) and Kv-ArcLight-ST under CAG promoter in the primary visual cortex. Optical traces of cells indicated with white circles are shown in **b**. Scale bar, 25 μm. (**b, c**) Fluorescence change of SEP A227D and Kv-ArcLight-ST (**b**) and stimulus-triggered average over 20 visual stimuli (**c**). Individual trials are shown with gray. Arrowheads indicate the timing of visual stimuli with flash light for 10 ms. (**d, e**) Peak fluorescence change (**d**) and SNR (**e**) of cellular response to visual stimuli. Mann-Whitney test was used for statistical analysis. *n* = 5 cells from 2 mice in each condition. Two-photon imaging was performed in anaesthetized mice with isoflurane (1.5% v/v). Excitation wavelength was 940 nm, and excitation power was 130 ~ 150 mW (0.50 ~ 0.61 μW/pixel) at the imaging plane. Visual response was recorded 20 times in each cell or field of view. On each box in (d) and (e), the central line represents median, and the bottom and the top edges of the box represent 25th and 75th percentile, respectively. The lower and the upper whiskers extend to the minimum and the maximum data points, respectively.

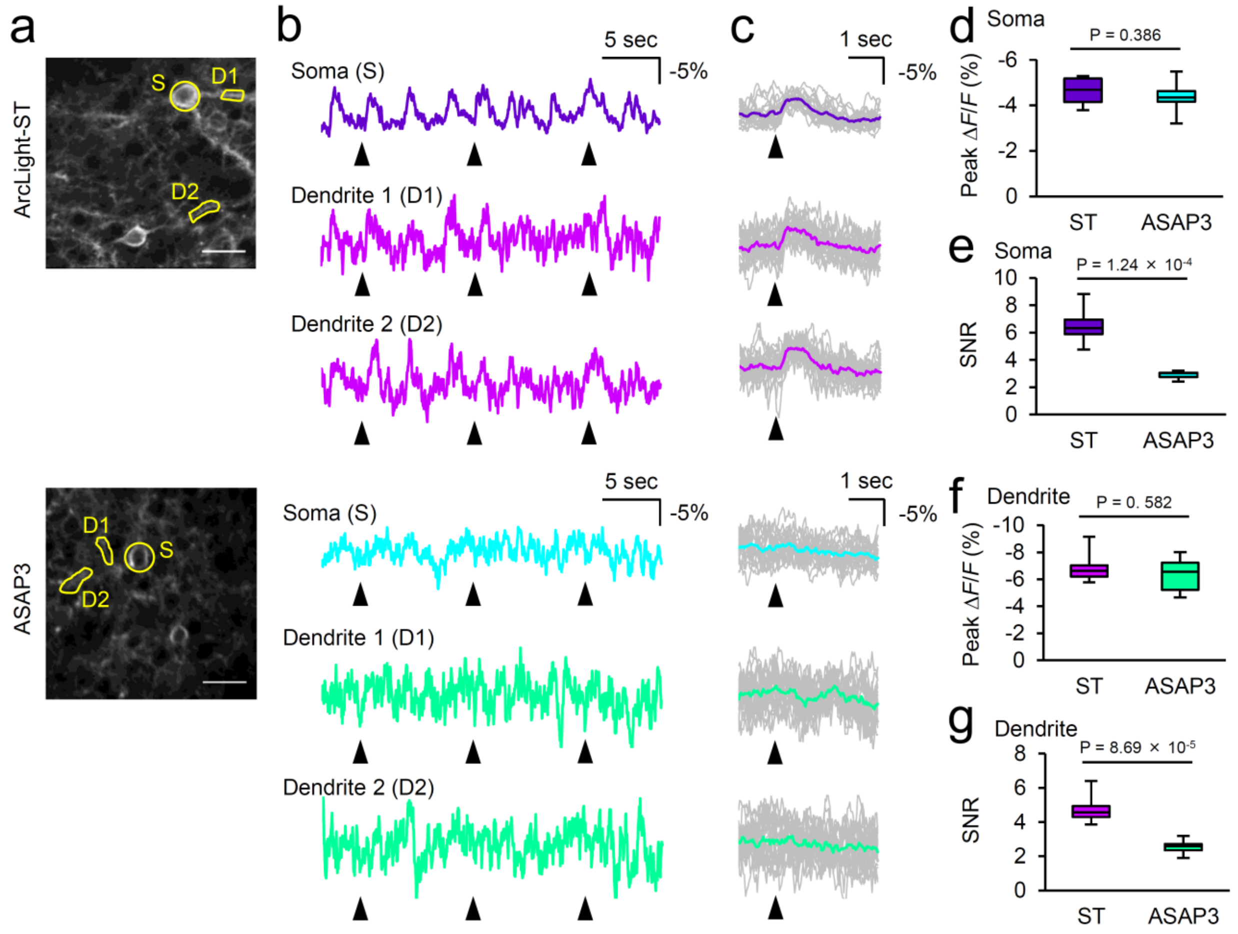

Supplementary Figure 7 | Performance of ArcLight-ST and ASAP3 with two-photon imaging *in vivo*. (**a**) Two-photon image of layer 2/3 neurons expressing ArcLight-ST and ASAP3 under CAG promoter in V1. Optical traces of cells indicated with yellow area are shown in **b**. Scale bar, 25 μm. (**b, c**) Fluorescence change of ArcLight-ST and ASAP3 (**b**) and stimulus-triggered average over 20 visual stimuli (**c**). Individual trials are shown with gray. Arrowheads indicate the timing of visual stimuli with flash light for 10 ms. (**d-g**) Peak fluorescence change in soma (**d**) and dendrite (**f**) and SNR in soma (**e**) and dendrite (**g**) of cellular response to visual stimuli. Mann-Whitney test was used for statistical analysis. *n* = 11 cells (ArcLight-ST), 10 (ASAP3) (d, e), 12 dendrites (ArcLight-ST), 10 (ASAP3) (f, g). Two mice were examined in each condition. Two-photon imaging was performed in anaesthetized mice with isoflurane (1.5% v/v). Excitation wavelength was 920 nm (ASAP3) or 940 nm (ArcLight-ST), and excitation power was 130 ~ 150 mW (0.50 ~ 0.61 μW/pixel) at the imaging plane. Visual response was recorded 20 times in each cell or field of view. On each box in (d) ~ (g), the central line represents median, and the bottom and the top edges of the box represent 25th and 75th percentile, respectively. The lower and the upper whiskers extend to the minimum and the maximum data points, respectively.

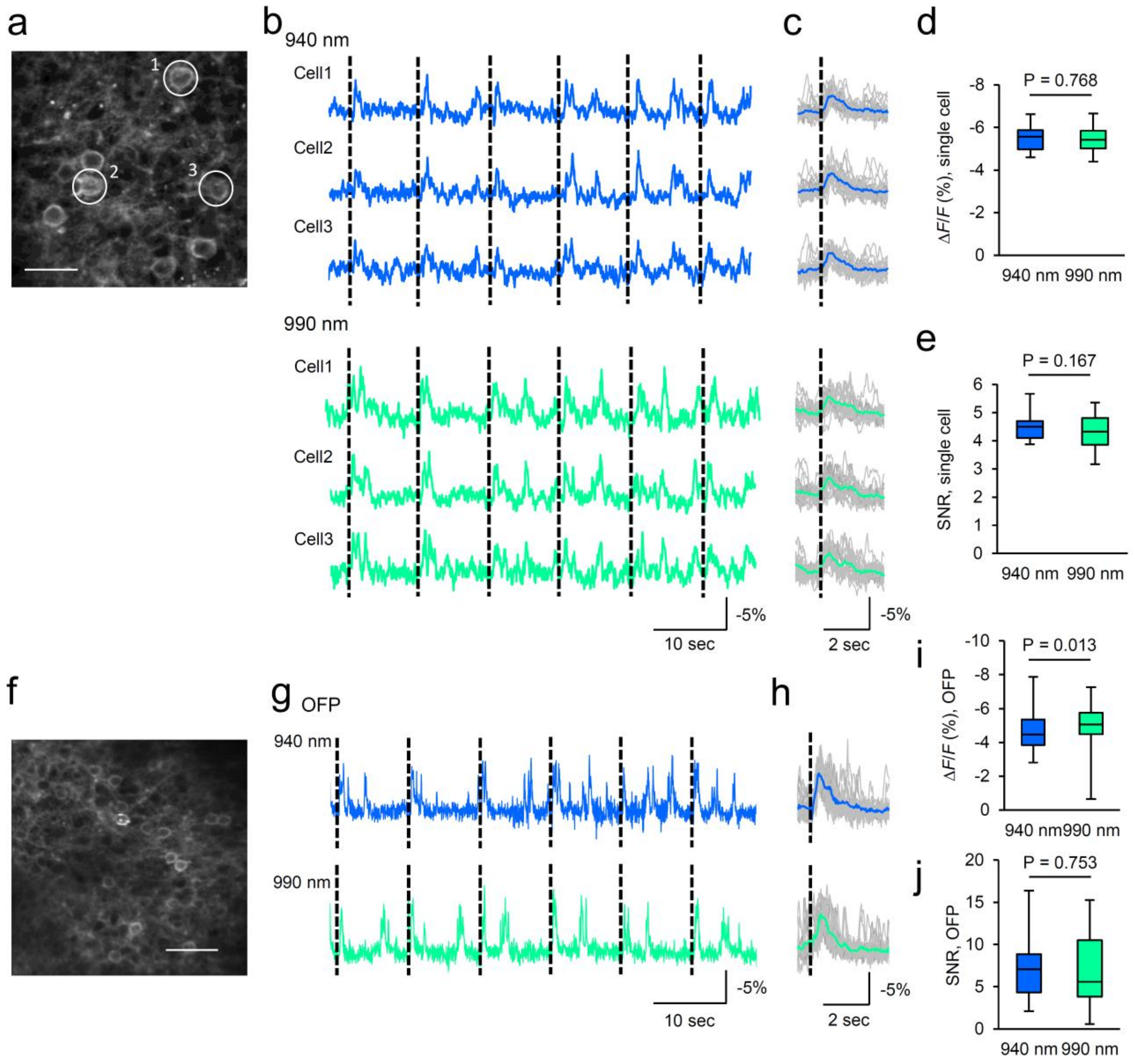

Supplementary Figure 8 | Comparison of different excitation wavelength for two-photon voltage imaging with Kv-ArcLight-ST *in vivo*. (**a**) Two-photon image of layer 2/3 neurons expressing Kv-ArcLight-ST in the primary visual cortex. White circles and numbers indicate cells whose optical traces are shown in (**b**) and (**c**). Scale bar, 25 μm. (**b, c**) Fluorescence change of Kv-ArcLight-ST (**b**) and stimulus-triggered average over 20 visual stimuli (**c**) with excitation at 940 nm and 990 nm. Individual trials are shown with gray. Dashed lines indicate the timing of visual stimuli of 10 ms with flash light. (**d, e**) Peak fluorescence change (**d**) and SNR (**e**) in response to visual stimuli. Mann-Whitney test was used for statistical analysis. n = 28 cells (940 nm), 29 (990 nm). Three mice were examined in each condition. (**f**) Two-photon image of layer 2/3 neurons expressing Kv-ArcLight-ST in the primary visual cortex for recording of OFP. Scale bar, 50 μm. (**g, h**) Fluorescence change of Kv-ArcLight-ST (**g**) and stimulus-triggered average over 20 visual stimuli (**h**) with excitation at 940 nm and 990 nm. Individual trials are shown with gray. Dashed lines indicate the timing of visual stimuli of 10 ms with flash light. (**i, j**) Peak fluorescence change (**i**) and SNR (**j**) in response to visual stimuli. n = 60 events (940 nm, optical field), 60 (990 nm, optical field). Mann-Whitney test was used for statistical analysis. Visual response was recorded 20 times in each cell or field of view. Two mice

were examined for each condition. Two-photon imaging was performed in anaesthetized mice with isoflurane (1.5% v/v). Excitation power was 130 ~ 160 mW (0.50 ~ 0.61 μW/pixel) (940 nm) or 150 ~ 180 mW (0.57 ~ 0.69 μW/pixel) (990 nm) at the imaging plane. On each box in (d), (e), (i) and (j), the central line represents median, and the bottom and the top edges of the box represent 25th and 75th percentile, respectively. The lower and the upper whiskers extend to the minimum and the maximum data points, respectively.

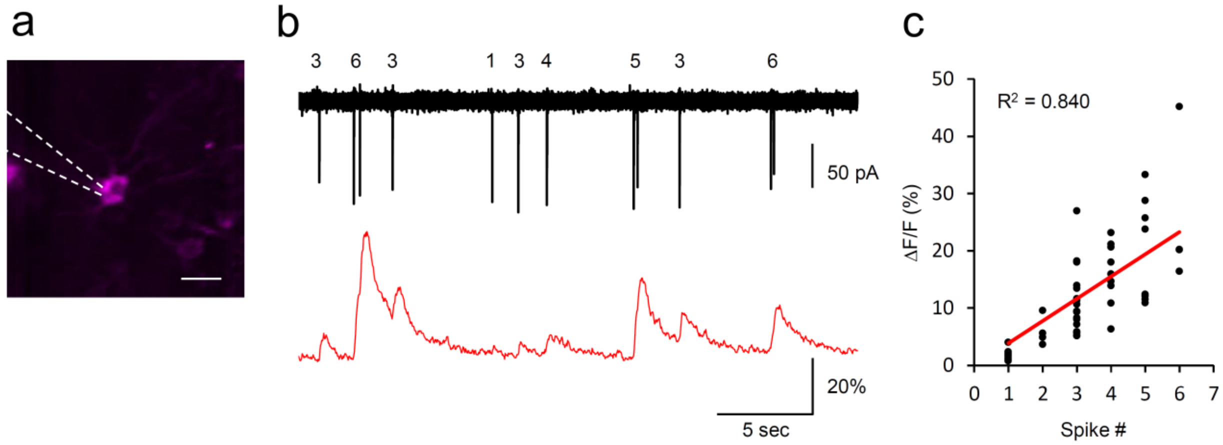

Supplementary Figure 9 | Performance of jRGECO1a with excitation at 990 nm *in vivo*. (**a**) Two-photon image of layer 2/3 neurons expressing jRGECO1a in the primary visual cortex. Dashed line indicates position of glass pipette. Scale bar, 25 μm. (**b**) Simultaneous cell-attached recording and two-photon Ca^{2+} imaging of spontaneous firing *in vivo*. An electrical trace is shown in black, and an optical trace is shown in red. Numbers above electrical trace indicate spike number. (**c**) Correlation between spike number and fluorescence change of jRGECO1a. Two cells from 2 mice were examined. Two-photon imaging was performed in anaesthetized mice with isoflurane (1.5% v/v). Excitation power was 150 ~ 180 mW (0.57 ~ 0.69 μW/pixel) (990 nm) at the imaging plane.

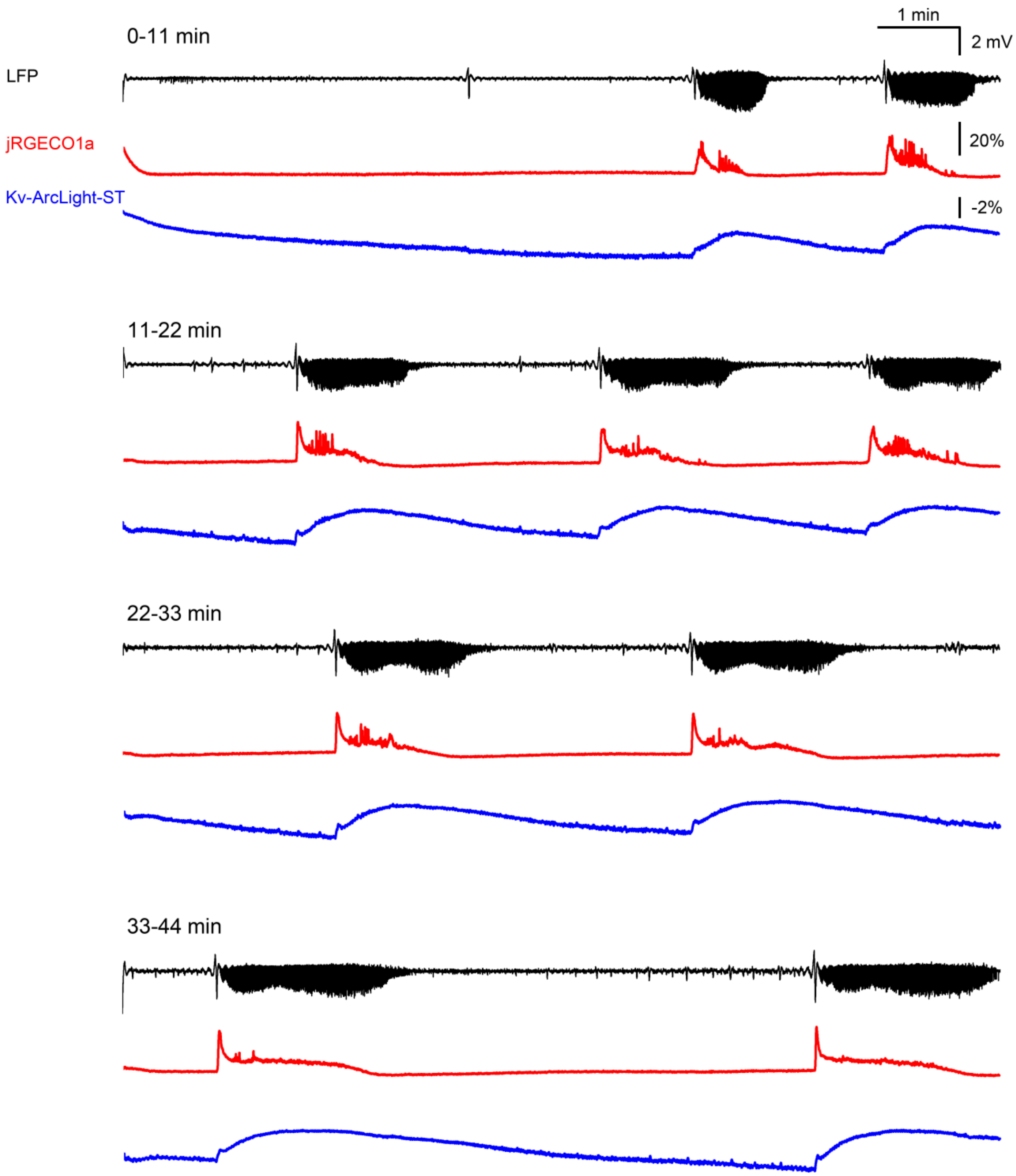

Supplementary Figure 10 | Long time scale two-photon imaging of sub- and suprathreshold events during epileptic conditions *in vivo*. Simultaneous calcium and voltage (optical field potential) imaging (propagation area) and LFP recording (seizure initiation site). Kv-ArcLight-ST and jRGECO1a were expressed under CAG promoter using *in utero* electroporation. Imaging was performed 150 ~ 300 μm below the surface (two-photon) in lightly anaesthetized mice (isoflurane, ~ 1.5% v/v). Excitation wavelength was 990 nm. Excitation power was 150 ~ 180 mW (0.57 ~ 0.69 μW/pixel) at the imaging plane.

		$\Delta F/F$ (%)	SNR	Rise time			Decay		
				τ_{fast} (ms)	τ_{slow} (ms)	Fast component (%)	τ_{fast} (ms)	τ_{slow} (ms)	Fast component (%)
20 mV depolarization	ArcLight-MT	-4.94 ± 0.81	4.25 ± 0.67	55.5 ± 5.3	796.8 ± 217.8	76.9 ± 6.6	50.2 ± 4.7	1542.9 ± 367.5	79.1 ± 4.1
(-70 to -50 mV)	ArcLight-ST	-7.72 ± 0.57	8.42 ± 1.86	81.1 ± 9.5	463.7 ± 64.4	73.6 ± 6.2	78.2 ± 9.9	1224.6 ± 296.0	79.9 ± 2.1
	Kv-ArcLight-ST	-8.38 ± 0.60	7.20 ± 0.68	61.0 ± 3.1	1005.0 ± 139.6	82.7 ± 1.9	59.5 ± 3.0	1625.3 ± 381.7	81.9 ± 1.3
100 mV depolarization	ArcLight-MT	-26.8 ± 2.1	26.23 ± 5.17	37.2 ± 3.6	163.1 ± 22.0	81.0 ± 10.1	45.6 ± 3.0	518.3 ± 119.6	90.8 ± 2.4
(-70 to +30 mV)	ArcLight-ST	-18.5 ± 1.8	13.39 ± 2.63	54.7 ± 7.1	214.6 ± 19.4	38.7 ± 11.8	113.4 ± 7.3	964.8 ± 160.8	88.2 ± 4.6
	Kv-ArcLight-ST	-15.4 ± 2.0	11.46 ± 1.70	120.6 ± 45.6	376.2 ± 79.1	69.0 ± 4.6	78.4 ± 9.0	1000.1 ± 311.0	86.0 ± 7.3
	ArcLight-MT	-7.04 ± 0.35	7.86 ± 0.32	10.7 ± 3.9	-	-	93.4 ± 14.1	-	-
Action potentials	ArcLight-ST	-3.77 ± 0.16	4.35 ± 0.24	-	-	-	-	-	-
	Kv-ArcLight-ST	-5.64 ± 0.36	3.76 ± 0.21	-	-	-	-	-	-

Supplementary Table 1 | Properties of ArcLight variants with one-photon imaging (Mean ± SEM is presented).
