# Supplementary material for: Simultaneous two-photon imaging of action potentials and subthreshold inputs in vivo
Source: Nat Commun. 2021 Dec 10;12:7229. doi: 10.1038/s41467-021-27444-9 (PMC8664861; doi:10.1038/s41467-021-27444-9)
Supplement: Supplementary file 3 — Reporting Summary [file 41467_2021_27444_MOESM3_ESM.pdf]

nature portfolio

Corresponding author(s): Yuki Bando

Last updated by author(s): 2021/10/29

Reporting Summary

Nature Portfolio wishes to improve the reproducibility of the work that we publish. This form provides structure for consistency and transparency in reporting. For further information on Nature Portfolio policies, see our Editorial Policies and the Editorial Policy Checklist.

Statistics

For all statistical analyses, confirm that the following items are present in the figure legend, table legend, main text, or Methods section.

n/a	Confirmed	
☐	☒	The exact sample size (n) for each experimental group/condition, given as a discrete number and unit of measurement
☐	☒	A statement on whether measurements were taken from distinct samples or whether the same sample was measured repeatedly
☐	☒	The statistical test(s) used AND whether they are one- or two-sided<br>*Only common tests should be described solely by name; describe more complex techniques in the Methods section.*
☒	☐	A description of all covariates tested
☒	☐	A description of any assumptions or corrections, such as tests of normality and adjustment for multiple comparisons
☐	☒	A full description of the statistical parameters including central tendency (e.g. means) or other basic estimates (e.g. regression coefficient) AND variation (e.g. standard deviation) or associated estimates of uncertainty (e.g. confidence intervals)
☐	☒	For null hypothesis testing, the test statistic (e.g. F, t, r) with confidence intervals, effect sizes, degrees of freedom and P value noted<br>*Give P values as exact values whenever suitable.*
☒	☐	For Bayesian analysis, information on the choice of priors and Markov chain Monte Carlo settings
☒	☐	For hierarchical and complex designs, identification of the appropriate level for tests and full reporting of outcomes
☒	☐	Estimates of effect sizes (e.g. Cohen's d, Pearson's r), indicating how they were calculated

Our web collection on statistics for biologists contains articles on many of the points above.

Software and code

Policy information about availability of computer code

Data collection	HC Image (one-photon imaging with sCMOS camera (Hamamatsu)), Multiclamp700B commander (electrophysiological recording (Axon Instruments)), PackIO (custom-made LabView-based software for electrophysiological recording), Prairie View 5.3 (two-photon imaging and electrophysiological recording (Bruker))
Data analysis	ImageJ 1.52a (NIH), MATLAB R2019b (Math Works), KyPlot5.0 (Kyenslab), Excel 365 (Microsoft)

For manuscripts utilizing custom algorithms or software that are central to the research but not yet described in published literature, software must be made available to editors and reviewers. We strongly encourage code deposition in a community repository (e.g. GitHub). See the Nature Portfolio guidelines for submitting code & software for further information.

Data

Policy information about availability of data

All manuscripts must include a data availability statement. This statement should provide the following information, where applicable:

- Accession codes, unique identifiers, or web links for publicly available datasets
- A description of any restrictions on data availability
- For clinical datasets or third party data, please ensure that the statement adheres to our policy

Source Data was provided in the Supplementary Information.

Field-specific reporting

Please select the one below that is the best fit for your research. If you are not sure, read the appropriate sections before making your selection.

☒ Life sciences ☐ Behavioural & social sciences ☐ Ecological, evolutionary & environmental sciences

For a reference copy of the document with all sections, see nature.com/documents/nr-reporting-summary-flat.pdf

Life sciences study design

All studies must disclose on these points even when the disclosure is negative.

Sample size	Statistical estimation of sample size was not performed. Sample size was determined from preliminary experiments and the sample size that is widely used in this field (Ref. 3-11).
Data exclusions	Mice were excluded from analysis if voltage or calcium probe was poorly expressed. In electrophysiological experiments, data was excluded from analysis if resting potential was greater than -50 mV or input resistance was smaller than 100 MOhm, based on the previous studies (Ref. 5, 11, Takeuchi et al., PLoS ONE, 2008).
Replication	For in vitro charachterization of ArcLight variants, experiments were performed with at least three batches of cultures, and showed similar results. Experiments in acute brain slices and in vivo were performed with at least two mice, and showed comparable results.
Randomization	Mice and cultured neurons were randomly assigned to each experimental group.
Blinding	Blinding was not performed in this study because one experimenter was responsible for all procedures. Data was collected in the equall conditions, and was analyzed automatically with the equal condition. Data was reproduced among multiple replicates.

Reporting for specific materials, systems and methods

We require information from authors about some types of materials, experimental systems and methods used in many studies. Here, indicate whether each material, system or method listed is relevant to your study. If you are not sure if a list item applies to your research, read the appropriate section before selecting a response.

Materials & experimental systems

n/a	Involved in the study	
☒	☐	Antibodies
☒	☐	Eukaryotic cell lines
☒	☐	Palaeontology and archaeology
☐	☒	Animals and other organisms
☒	☐	Human research participants
☒	☐	Clinical data
☒	☐	Dual use research of concern

Methods

n/a	Involved in the study	
☒	☐	ChIP-seq
☒	☐	Flow cytometry
☒	☐	MRI-based neuroimaging

Animals and other organisms

Policy information about studies involving animals; ARRIVE guidelines recommended for reporting animal research

Laboratory animals	Both sex of wild-type CD-1 mice (Charles River) were used. Age: E16 (in utero electroporation), E18 (primary neuronal culture), P11~14 (acute brain slices), P35~60 (in vivo experiments). Animal room was kept between 20 ~ 25 ℃, and between 40 ~ 60% humidity.
Wild animals	Wild animals were not used in this study.
Field-collected samples	Field-collected samples were not used in this study.
Ethics oversight	Experimental procedures were carried out in accordance with the guidelines for animal care and use of the U.S. National Institute of Health, Columbia University and U.S. Army Research Office. All protocols using animals were approved by Institutional Animal Care and Use Committees in Columbia University and Animal Care and Use Review Office in U.S. Army Research office.

Note that full information on the approval of the study protocol must also be provided in the manuscript.
